# Threefold Fermions, Weyl Points, and Superconductivity in the Mirror Symmetry Lacking Semiconductor TlCd_2_Te_4_

**DOI:** 10.3390/nano12040679

**Published:** 2022-02-18

**Authors:** Angus Huang, Chin-Hsuan Chen, Horng-Tay Jeng

**Affiliations:** 1 Department of Physics, National Tsing Hua University, Hsinchu 30013, Taiwan; rabbit4a9@gmail.com (A.H.); barry810929@gmail.com (C.-H.C.); 2 Department of Physics, National Cheng Kung University, Tainan 70101, Taiwan; 3 Physics Division, National Center for Theoretical Sciences, Hsinchu 30013, Taiwan; 4 Institute of Physics, Academia Sinica, Taipei 11529, Taiwan

**Keywords:** threefold fermions, Weyl points, superconductivity

## Abstract

The topological phase transition and exotic quasiparticles in materials have attracted much attention because of their potential in spintronics and mimic of elementary particles. Especially, great research interest has been paid to search for the Weyl fermions in solid-state physics. By using first-principles calculations, we predict that the multinary semiconductor alloy $\mathrm{Tl}{\mathrm{Cd}}_{2}{\mathrm{Te}}_{4}$ exhibits threefold fermions and nodal-line fermions, which are protected by the $S_{4}$ improper rotational symmetry. Moreover, owing to the lack of inversion and mirror symmetries, the threefold fermions split into Weyl fermions when the spin-orbit coupling is included. The chiral charge of Weyl points and the $Z_{2}$ time-reversal topological invariant are investigated. The topological surface states, spin texture, and electron-phonon coupling analysis are presented. Our study demonstrates $\mathrm{Tl}{\mathrm{Cd}}_{2}{\mathrm{Te}}_{4}$ as a good platform to understand topological phase transitions as well as possible coexistance of topological Weyl semimetal and superconductivity in one single material.

## 1. Introduction

Since the research of topological materials, the quasiparticles in solid-state materials have provided a tabletop platform to imitate the particles in high-energy physics. Several kinds of particles have been discovered by experiments or theoretical predictions, such as the Dirac fermions [1,2,3], Weyl fermions [4,5], and spin-3/2 Rarita-Schwinger-Weyl (RSW) fermions [6]. Furthermore, some quasiparticles beyond particle physics have also been demonstrated in materials, for example, type-II Dirac/Weyl fermions [7,8], nodal-line [9,10], quadratic or cubic Weyl fermions [11,12], hourglass fermions [13,14,15], and high-fold fermions [6,16,17,18,19,20,21]. The topological phase transitions also give a route to study the interaction between elementary particles.

In particular, Weyl fermions have attracted much interest because of the predictions but non-observed in elementary particle physics. It has been theoretically predicted [8] and experimentally observed [22] that Dirac fermions could split into Weyl fermions. However, the phase transitions of Weyl fermions to other exotic fermions, such as the threefold fermions (TFFs, also known as three-component fermions [18], triple-point fermions [21]), have not been discovered yet.

In this work, using first-principles calculations based on density functional theory (DFT), we study the multinary semiconductor alloy material $\mathrm{Tl}{\mathrm{Cd}}_{2}{\mathrm{Te}}_{4}$, which has been synthesized by previous experiments [23]. Two TFFs and nodal-line fermions in $\mathrm{Tl}{\mathrm{Cd}}_{2}{\mathrm{Te}}_{4}$ stemmed from the $S_{4}$ improper rotational symmetry are demonstrated. When spin-orbit coupling is included, owing to the lack of inversion and mirror symmetries, a topological phase transition occurs and each TFFs split into four Weyl points (WPs) with chiral charge C=±1. The $Z_{2}$ topological invariant $v_{Z_{2}}=1$ are also proved by Wilson loops (WLs) analysis. We also present the topological surface states (TSSs) of $\mathrm{Tl}{\mathrm{Cd}}_{2}{\mathrm{Te}}_{4}$, which may be observed in future experiments. Finally, we also show the stable phonon bands of $\mathrm{Tl}{\mathrm{Cd}}_{2}{\mathrm{Te}}_{4}$ as well as the superconductivity of $T_{C}=3.8\;K$ after electron doping of 0.5 e/f.u.

## 2. Method

The first-principles calculations are carried out using Vienna Ab-initio Simulation Package (VASP) [24,25] utilizing the Ceperly-Alder (CA) type exchange-correlation functional and all-electron projected augmented wave (PAW) method within the framework of local density approximation (LDA) [26] based on density functional theory (DFT). The 12×12×12k−grids over the first Brillouin zone (BZ) with the energy cut-off of 400 eV are used in the self-consistent calculations. The lattice structure and space group of $\mathrm{Tl}{\mathrm{Cd}}_{2}{\mathrm{Te}}_{4}$ is obtained from previous experiments [23]. Then, the lattice parameters and positions of ions are optimized until the residual atomic forces are less than 0.03eV/Å. The tight-binding Hamiltonian with Wannier basis is constructed from Te−p orbitals using the *vasp2wannier90* interface [27]. For topological properties, such as the Wilson loop (WL) [28,29], $Z_{2}$ topological invariant, and chiral charge, simulations are performed using the formula [30]

(1) $$\phi_{m}=-\mathrm{im}\log\sum_{i}\langle k_{i}\left|k_{i+1}\right.\rangle .$$

Here $\phi_{m}$ and $|k_{i}\rangle$ are the Berry phase and the wave functions of the *m*th band and *i*th k-point, respectively. The semi-infinite Green functions of surface states are simulated using the Sancho-Rubio method [31]. The quasiparticle interference (QPI) χ(q,E) are computed through the spin-dependent joint density of states (JDOS) [32,33]

(2) $$\chi \left(q,E\right)=\sum_{k,i}A^{i}\left(k,E\right)A^{i}\left(k+q,E\right)$$

with

(3) $$A^{i}\left(k,E\right)\equiv \langle S_{i}\left(k,E\right)\rangle =-\mathrm{im}\;\mathrm{tr}\left(G\left(k,E\right)\sigma^{i}\right).$$

Here $A^{i}$, $S_{i}$, and *G* are the spin-dependent spectrum, spin operator, and Green functions of surface states, respectively. $\sigma^{i}$ (i=0,1,2,3) are the identity matrix (i=0) and Pauli matrix (i=1,2,3). The 400×400k−mesh are used in QPI simulations.

For phonon and electron-phonon coupling simulations, the density functional perturbation theory (DFPT) calculations are performed using the quantum espresso package [34] over the k−mesh (q−mesh) of 32×32×32 (4×4×4). Superconducting $T_{C}$ is estimated by the Allen-Dynes modified McMillan formula [35]

(4) $$T_{C}=f_{1}f_{2}\frac{{\bar{\omega }}_{\log}}{1.2}\exp\left(\frac{1.04(1+\lambda )}{\mu^{*}\left(1+0.62\lambda \right)-\lambda }\right)$$

and Eliashberg function $\alpha^{2}F\left(\omega \right)$

(5) $$\alpha^{2}F\left(\omega \right)=\frac{1}{2}\int_{\mathrm{BZ}}\lambda_{qv}\omega_{qv}\delta \left(\omega -\omega_{qv}\right)\;dq.$$

Here $\omega_{qv}$ (${\bar{\omega }}_{\log}$) is the phonon band (logarithmic average phonon) frequency. The λ ($\lambda_{qv}$) is the electron-phonon coupling strength (electron-phonon coupling coefficient of each band), from the DFPT calculations. Furthermore, we calculate the integral of $\lambda_{qv}$, $\lambda \left(\omega \right)\equiv \int^{\omega }\sum_{qv}\lambda_{qv}dq$. The $\mu^{*}$ is the effective Coulomb repulsion, we choose $\mu^{*}=0.1$ in this work as is commonly used in superconductivity calculations. $f_{1}$ and $f_{2}$ are the same as those given by Allen and Dynes [35].

## 3. Electronic Structures

**Figure 1:**
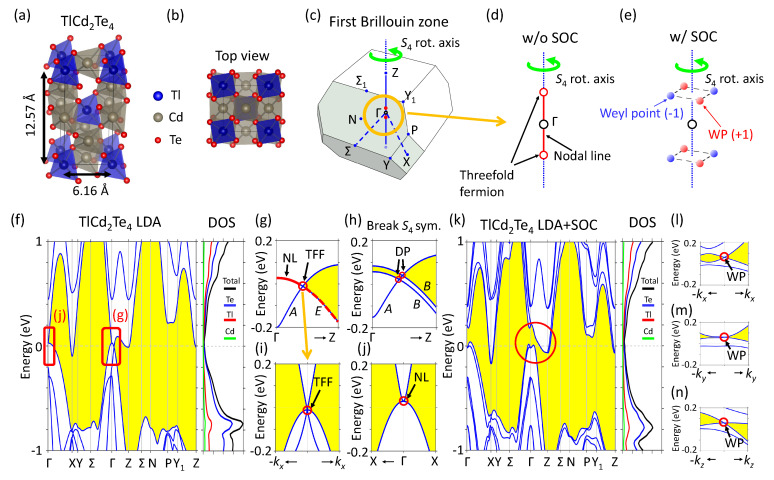
The lattice and band structure of $\mathrm{Tl}{\mathrm{Cd}}_{2}{\mathrm{Te}}_{4}$. (**a**) The lattice structure and parameters of $\mathrm{Tl}{\mathrm{Cd}}_{2}{\mathrm{Te}}_{4}$. (**b**) The top view of $\mathrm{Tl}{\mathrm{Cd}}_{2}{\mathrm{Te}}_{4}$. (**c**) The first Brillouin zone of $\mathrm{Tl}{\mathrm{Cd}}_{2}{\mathrm{Te}}_{4}$. The red points indicate the threefold fermion. (**d**) The sketch of threefold fermions and nodal-line in the Brillouin zone. (**e**) The 2 threefold fermions in (**d**) split into 8 Weyl points due to SOC. (**f**) The band structure and DOS of $\mathrm{Tl}{\mathrm{Cd}}_{2}{\mathrm{Te}}_{4}$. (**g**) The zoom-in of threefold fermion (TFF). (**h**) The band structures with breaking $S_{4}$ symmetry. (**i**) The TFF in the $k_{x}$ direction. (**j**) the nodal-line at Γ point in the XΓX direction. (**k**) The band structure and DOS with SOC. (**l**–**n**) The band structure of Weyl point (WP) along different directions.

The multinary semiconductor alloy $\mathrm{Tl}{\mathrm{Cd}}_{2}{\mathrm{Te}}_{4}$ has been synthesized by previous experiments [23]. Based on experimental results, $\mathrm{Tl}{\mathrm{Cd}}_{2}{\mathrm{Te}}_{4}$ has space group $I\bar{4}$ (#82) with thiogallate structure (defect-chalcopyrite structure, also presented in ordered-vacancy compounds of $AB_{2}X_{4}$ [36]), which lacks the inversion symmetry while keeps an $S_{4}$ improper rotational symmetry in the *z*-direction. Unlike the well-known Weyl semimetals such as TaAs and $1T^{d}-W{\mathrm{Te}}_{2}$, $\mathrm{Tl}{\mathrm{Cd}}_{2}{\mathrm{Te}}_{4}$, however, has no mirror symmetry, which makes it a special case in the Weyl family. The lattice structure and atomic positions of $\mathrm{Tl}{\mathrm{Cd}}_{2}{\mathrm{Te}}_{4}$ have been geometrically relaxed in our DFT calculations. The optimized lattice constants are a=6.16Å and c=12.57Å. Each Tl/Cd ion is surrounded by four Te ions, forming $\mathrm{Tl}{\mathrm{Te}}_{4}$/$\mathrm{Cd}{\mathrm{Te}}_{4}$ tetrahedrons, as shown in Figure 1a. All $\mathrm{Tl}{\mathrm{Te}}_{4}$/$\mathrm{Cd}{\mathrm{Te}}_{4}$ tetrahedrons rotate a small angle along the *z*-axis as presented in Figure 1b, which breaks the inversion symmetry and mirror symmetry of $\mathrm{Tl}{\mathrm{Cd}}_{2}{\mathrm{Te}}_{4}$.

The band structure of $\mathrm{Tl}{\mathrm{Cd}}_{2}{\mathrm{Te}}_{4}$ along high-symmetry lines in Brillouin zone (BZ) (Figure 1c) calculated using LDA is shown in Figure 1f. The bands near the Fermi level are dominated by Te−p orbitals. There exists a continuous energy band gap as highlighted by the yellow region with only two band crossing points around the Γ-point as indicated by the red rectangles. The zoom-in pictures of these band crossings are presented in Figure 1g,j. The band crossing (red circle in Figure 1g) between the Γ and Z is composed of a single band (blue line) with a double degenerate nodal-line band (red-on-blue line in Figure 1g). To verify if this crossing is protected by $S_{4}$ symmetry, we studied the character of this eigenstate from the tight-binding model with Wannier orbitals. The symmetry analysis demonstrates that the nodal-line and single band have eigenvalues of ±i and +1 in $S_{4}$ symmetry denoted by *E* and *A*, respectively, in the group representations [37], as shown in Figure 1g. Their different group representations assure that this band crossing is $S_{4}$ symmetry protected TFF. Moreover, owing to the $S_{4}$ symmetry, the TFF come in pairs: one along $+k_{z}$ and the other along $-k_{z}$, with the nodal-line connecting them as sketched in Figure 1d. The $S_{4}$ protected nodal-line also presents another band crossing in XΓX, as shown in Figure 1j. Owing to the lack of $S_{4}$ symmetry away from ΓZ axis, the double degenerate nodal-line at TFF in the ΓZ axis splits into two bands along the $k_{x}-$direction as presented in Figure 1i. On the other hand, we break the $S_{4}$ symmetry by artificially moving some ions while keeping the $C_{2}$ symmetry. Without the $S_{4}$ symmetry, the nodal-line splits into two bands with eigenvalues of −1 in the $C_{2}$ symmetry. The TFF thus splits into two Dirac points as demonstrated in Figure 1h. This result, combined with previous group representation analysis, clearly proves that both TFFs and nodal-line are protected by $S_{4}$ symmetry.

To study the topological properties, the spin-orbit coupling (SOC) is included in the calculations of band structures as illustrated in Figure 1k. As can be seen, SOC removes all the band crossings from the high-symmetry lines, as highlighted by the red circle in Figure 1k. In comparison with Figure 1f, SOC also opens up an energy gap of ∼0.2 eV around the Γ-point, which serves as a rough estimation of the strength of SOC in $\mathrm{Tl}{\mathrm{Cd}}_{2}{\mathrm{Te}}_{4}$. However, the lack of inversion symmetry implies that Weyl points (WPs) could exist in non-high symmetry k−points. We use the tight-binding model with Wannier orbitals to find the band crossings in the full first Brillouin zone. Eight WPs in total are found as shown in Figure 1e. One of the WPs locates at $k_{\mathrm{WP}1}=\left(0.042,0.012,0.120\right)\;{Å}^{-1}$, and the other seven WPs are symmetric to the first one. The evolution from TFF to WPs due to SOC can be seen in comparison with Figure 1d,e: SOC splits each TFF into four WPs. DFT band structures of one selected WP are shown in Figure 1l–n, which present strong anisotropy in band structures. Furthermore, the linear band dispersions imply the WPs carry the chiral charge C=±1.

## 4. Topological Phases, Surface State, and QPI

**Figure 2:**
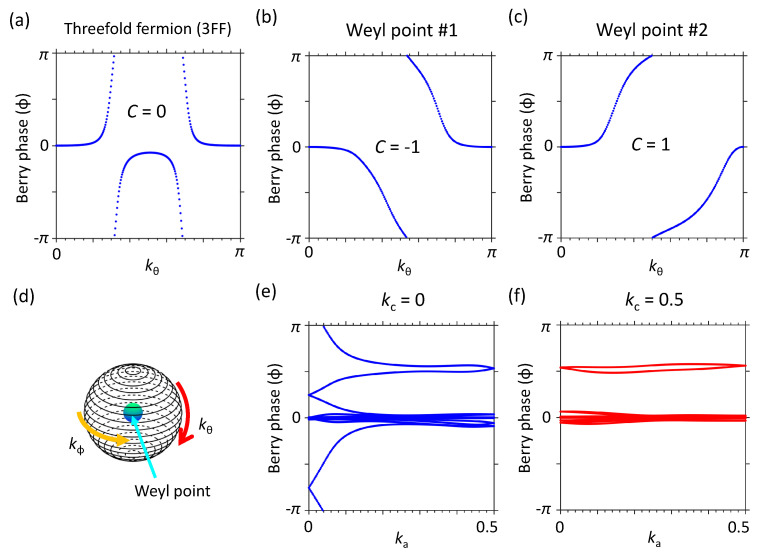
(**a**) The Wilson loop (WL) of TFF of $\mathrm{Tl}{\mathrm{Cd}}_{2}{\mathrm{Te}}_{4}$. (**b**,**c**) The WL at two different WPs (WPs). (**d**) The sphere surrounding the crossing point for simulating the WL. (**e**,**f**) The WL of $Z_{2}$ topological invariant.

The Berry phase and Wilson loop (WL) of $\mathrm{Tl}{\mathrm{Cd}}_{2}{\mathrm{Te}}_{4}$ shown in Figure 2 are calculated on the sphere surrounding the crossing points illustrated in Figure 2d. Previous studies of TFF with SOC present the topological properties with Chern number C=2 [38]. For $\mathrm{Tl}{\mathrm{Cd}}_{2}{\mathrm{Te}}_{4}$, the WLs of TFFs present zero times winding without SOC, that is C=0 as shown in Figure 2a. Nevertheless, the Berry phase present a sharp change in $k_{\theta }≃0.3\pi$ and $k_{\theta }≃0.7\pi$ with the opposite-sign, which implies the non-zero Berry curvature on the sphere and topological invariants could be induced when the SOC is included. For the Weyl semimetals with mirror symmetry, such as TaAs and $1T^{d}-W{\mathrm{Te}}_{2}$, the WPs are produced in pairs by SOC on both sides of the mirror symmetry planes. However, owing to the lack of mirror symmetry, one TFF splits into four WPs with different chiral charges in $\mathrm{Tl}{\mathrm{Cd}}_{2}{\mathrm{Te}}_{4}$. The chiral charge (C=±1) of different WPs, from the WL results, are shown in Figure 2b,c. To confirm the topological properties of $\mathrm{Tl}{\mathrm{Cd}}_{2}{\mathrm{Te}}_{4}$, the $Z_{2}$ topological invariant is provided in Figure 2e,f. The WLs show different winding numbers of 1 and 0 in different plane $k_{c}=0$ and $k_{c}=0.5$. This WLs result shows that $\mathrm{Tl}{\mathrm{Cd}}_{2}{\mathrm{Te}}_{4}$ also exhibits the non-trivial strong topological insulator property $v_{Z_{2}}=1$. From our knowledge, this topological phase transition from TFF to Weyl semimetal induced by SOC has not been reported to date.

**Figure 3:**
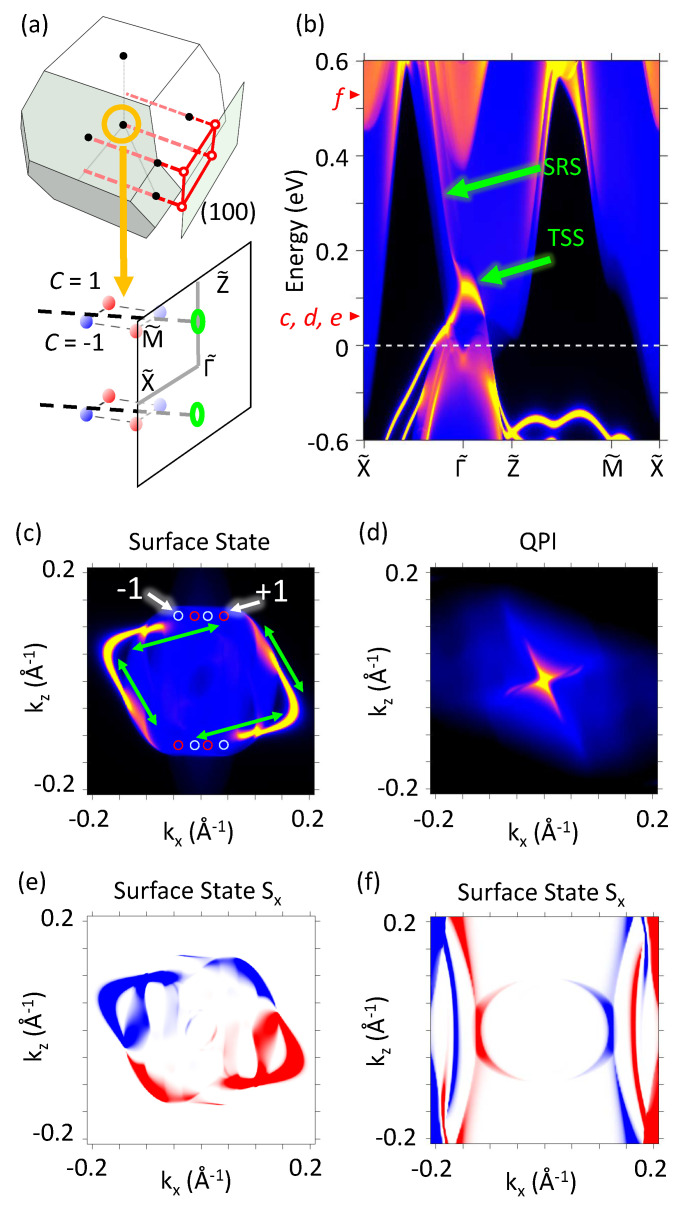
(**a**) The Brillouin zone and WPs projected onto the (100) surface. (**b**) The surface states calculated from semi-infinite Green function method. The small red arrows on the energy axis show the energies of subfigures (**c**–**f**). (**c**) The two-dimensional contour of BZ at E=0.06 eV on (100). The red (light blue) circles indicate the projection of Weyl point with chiral charge +1 (−1). (**d**) Cross-shaped QPI. (**e**) $\langle S_{x}\rangle$ of TSSs at E=0.06 eV. (**f**) $\langle S_{x}\rangle$ of SRSs at E=0.52 eV.

The WPs with different chiral charges presented in Figure 3a are close to each other and covered by bulk bands, which makes the WPs barely visible. The topological surface states (TSS) calculated using the semi-infinite slab Green function simulations for (100) surface are presented in Figure 3b. As highlighted by the short green arrow, the TSSs connect the valence bands with the conduction bands, demonstrating the $Z_{2}$ topological invariant behavior. Figure 3c shows the two-dimensional contour of TSSs at the same energy as that of the Weyl points (E=0.06eV). The TSSs connect the WPs and then extend into the bulk bands as surface resonance states (SRSs). Two parallelogram-shaped Fermi arcs (TSSs) connecting two WPs with different chiral charge can be seen. We have also performed the QPI simulations as shown in Figure 3d. The correlations (green arrows in Figure 3c) between parallelogram-shaped topological surface states forms a cross-shaped QPI, which is different from the common circle-shaped QPI. The spin textures of TSSs $\langle S_{i}\rangle =A^{i}\left(k,E\right)$ obtained from Equation (3) are shown in Figure 3e. Owing to the SOC, the spin of SSs rotate around the $\tilde{\Gamma }$. Moreover, we also show the spin textures of the SRSs at E=0.52eV in Figure 3f, which corresponds to the electron doping of 0.5e per formula unit (0.5e/f.u.).

## 5. Phonon Band and Superconductivity

**Figure 4:**
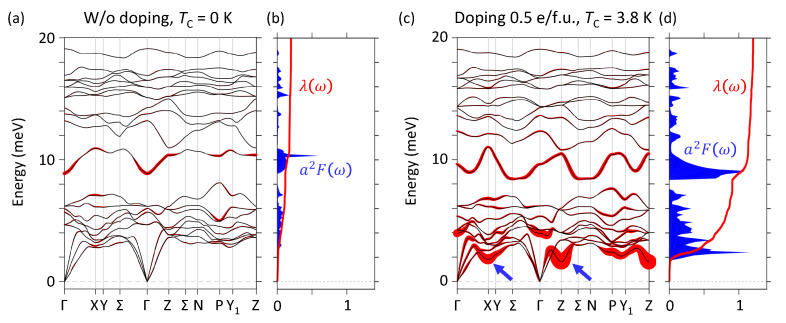
(**a**) The phonon band structure and electron-phonon coupling strength (red circle) of pristine $\mathrm{Tl}{\mathrm{Cd}}_{2}{\mathrm{Te}}_{4}$. (**b**) The electron-phonon coupling strength λ and Eliashberg function $\alpha^{2}F$ of pristine $\mathrm{Tl}{\mathrm{Cd}}_{2}{\mathrm{Te}}_{4}$. (**c**) The phonon band structure and electron-phonon coupling strength of 0.5 e/f.u. electron doped $\mathrm{Tl}{\mathrm{Cd}}_{2}{\mathrm{Te}}_{4}$. (**d**) The corresponding λ and $\alpha^{2}F$ of 0.5 e/f.u. electron doped $\mathrm{Tl}{\mathrm{Cd}}_{2}{\mathrm{Te}}_{4}$.

The phonon band structure and electron-phonon coupling strength λ of pristine $\mathrm{Tl}{\mathrm{Cd}}_{2}{\mathrm{Te}}_{4}$ and 0.5 e/f.u. electron doped $\mathrm{Tl}{\mathrm{Cd}}_{2}{\mathrm{Te}}_{4}$ are studied by using DFPT calculations. The phonon bands, λ(ω), and Eliashberg function are shown in Figure 4a,b. All the phonon bands are well behaved without imaginary phonon modes, showing that the structure of $\mathrm{Tl}{\mathrm{Cd}}_{2}{\mathrm{Te}}_{4}$ is stable. The electron-phonon coupling strength λ=0.18 presents the non-superconductivity in pristine $\mathrm{Tl}{\mathrm{Cd}}_{2}{\mathrm{Te}}_{4}$. However, after doping electrons of 0.5 e/f.u. as shown in Figure 4c, Kohn-anomalies highlighted by blue arrows are thus induced at X and Z points with strong electron-phonon coupling strength. It can be seen that the strongly enhanced electron-phonon coupling is significantly correlated with the softened phonon modes with relatively lower phonon energies forming the Kohn-anomalies. This is presumably due to the raised Fermi level up to 0.52 eV which may results in possible Fermi nesting among the Fermi pockets originated from the multi-valleys around 0.5 eV (Figure 1f,k). Moreover, the superconductivity emerges with $T_{C}=3.8\;K$ due to the highly raised λ=0.94. The result shows that suitable electron doping in $\mathrm{Tl}{\mathrm{Cd}}_{2}{\mathrm{Te}}_{4}$ could lead to a good platform for studying the superconductivity and topological Weyl semimetal coexisted system.

## 6. Discussion and Conclusions

Previous DFT works have demonstrated the mirror symmetry lacking Weyl semimetal SrSi${}_{2}$ [11]. Also, the topological phase transitions from TFFs to WPs have been predicted in BaAgAs by artificial breaking the inversion symmetry [39]. However, the topological phase transitions from TFFs to WPs induced by SOC demonstrated in our work have not been reported to date. Thus $\mathrm{Tl}{\mathrm{Cd}}_{2}{\mathrm{Te}}_{4}$ serves as a prototype material for studying the evolution of high-fold fermions beyond previous researches.

We note that because the defect is conceded as changing the electron numbers in the calculations, it has small influence to $\mathrm{Tl}{\mathrm{Cd}}_{2}{\mathrm{Te}}_{4}$ only. For real cases, doping might break more symmetry in $\mathrm{Tl}{\mathrm{Cd}}_{2}{\mathrm{Te}}_{4}$. However, owing that these Weyl points are not protected by the crystal symmetries, the topological properties of $\mathrm{Tl}{\mathrm{Cd}}_{2}{\mathrm{Te}}_{4}$ will not be changed by doping.

The robustness of topological properties and phase transaction are also important issues. We have further examined the strain effect on the topological properties of $\mathrm{Tl}{\mathrm{Cd}}_{2}{\mathrm{Te}}_{4}$ by applying uniform strain of −2% and −5%, which correspond to pressure of 2 GPa and 5.5 GPa, respectively. In both cases, the TFF and Weyl fermions move slightly in k-space with the topological properties kept unchanged. Thus the topological properties of $\mathrm{Tl}{\mathrm{Cd}}_{2}{\mathrm{Te}}_{4}$ are robust against strain effect.

In conclusion, using first-principle simulations, we study the topological properties and invariants in $\mathrm{Tl}{\mathrm{Cd}}_{2}{\mathrm{Te}}_{4}$ in this work. We show that the lacking of inversion and mirror symmetries in $\mathrm{Tl}{\mathrm{Cd}}_{2}{\mathrm{Te}}_{4}$ leads to the exotic topological phase. The splitting of threefold fermions (TFFs) into Weyl points (WPs) induced by SOC are demonstrated. The topological invariants, such as chiral charge and $Z_{2}$, and Fermi arc states have been studied in detail. Moreover, the phonon band structures and superconductivity with $T_{C}=3.8$ K for electron doped $\mathrm{Tl}{\mathrm{Cd}}_{2}{\mathrm{Te}}_{4}$ are also obtained by using DFPT. Our study sheds lights to a new evolution of topological phase transitions, as well as the possible coexistance of superconductivity and topological Weyl semimetal in one single material.

## Data Availability

Not applicable.
